# Rapid screening of copy number variations in *STR*C by droplet digital PCR in patients with mild-to-moderate hearing loss

**DOI:** 10.1038/s41439-019-0075-5

**Published:** 2019-08-30

**Authors:** Taku Ito, Yoshiyuki Kawashima, Taro Fujikawa, Keiji Honda, Ayane Makabe, Ken Kitamura, Takeshi Tsutsumi

**Affiliations:** 1 Department of Otorhinolaryngology, Tokyo Medical and Dental University, Tokyo, Japan; 2 Department of Otorhinolaryngology, Head and Neck Surgery, Chigasaki Chuo Hospital, Chigasaki, Japan

**Keywords:** Genetics research, Neurodegenerative diseases

## Abstract

Copy number variations (CNVs) are commonly reported in *STRC*, the causal gene for DFNB16. Various techniques are used clinically for CNV detection, and droplet digital PCR (ddPCR) provides highly precise absolute quantification of DNA copy number. We aimed to validate the feasibility and efficiency of ddPCR in combination with long-range PCR (LR-PCR) in identifying CNVs and mutations in *STRC*. Additionally, we determined the frequency of CNVs and mutations in *STRC* in Japanese patients with mild-to-moderate hearing loss. We evaluated 84 unrelated Japanese patients with mild-to-moderate bilateral idiopathic or autosomal recessive nonsyndromic sensorineural hearing loss. The ratio of *STRC* copy number to the copy number of the internal control *RPP30* ranged from 0.949 to 1.009 (0.989 ± 0.017) in 77 patients; it ranged from 0.484 to 0.538 (0.509 ± 0.024) in five patients and was 0.000 in two patients, indicating heterozygous and homozygous deletions, respectively. The copy number deletion prevalence rates were 7.7% and 0.9% in the patients and healthy controls, respectively. In combination with LR-PCR, ddPCR revealed that at least three patients (3.6%) had *STRC*-related hearing loss. Detecting *STRC* CNVs by ddPCR was rapid, precise, and cost-effective and facilitated the identification of *STRC* CNVs.

## Hearing loss: Counting copies

Detecting a common genetic cause of hearing loss is now easier, thanks to a new method. Loss of one or both copies of *STRC*, a gene required for function of the sound-detecting hairs in the inner ear, can cause hearing loss. Compared to detecting mutations, counting gene copies had been very challenging. Taku Ito at Tokyo Medical and Dental University, Japan, and colleagues applied a new technology that divides samples into thousands of droplets, then measures the genetic information in each droplet, allowing precise gene copy counting. Studying samples from nearly 100 Japanese patients with hearing loss, they were able to determine that hearing loss in several patients was caused by deletion of one or both copies of *STRC*. This method will speed diagnosis of *STRC-*related hearing loss, and has potential for application to other disorders.

## Introduction

Copy number variations (CNVs), defined as the presence of variable copy numbers of specific DNA segments, can be associated with a wide range of disorders. The most recent study has estimated that CNVs constitute 4.8–9.5% of the human genome and comprise a greater fraction of nucleotides than single nucleotide variants (SNVs). Specifically, in bilateral sensorineural hearing loss (SNHL), CNVs were identified in ~20% of all deafness-causing genes and found in ~15% of patients undergoing genetic testing^1–3^. Until recently, real-time quantitative PCR (qPCR) assays and microarray hybridization were the two main methods used to determine CNVs in the genome. Conversely, droplet digital PCR (ddPCR) allows for high-precision absolute quantification of DNA copy number. During ddPCR, each sample is partitioned into several tens of thousands of nanoliter-sized droplets prior to amplification. PCR amplification proceeds to the reaction endpoint, which is followed by the determination of whether every droplet in a well is positive or negative for both the target and the reference. The fluorescence intensity in each droplet is measured, and those above the threshold are counted as positive, thus providing a digital yes/no result. ddPCR overcomes several limitations of qPCR and microarray techniques for CNV analysis.

*STRC* (MIM: 606440) encodes the extracellular structural protein stereocilin, which is necessary for proper hair cell function and is the causal gene for DFNB16^4,5^. Mutations in *STRC*, commonly reported as CNVs, were described mainly in association with bilateral mild-to-moderate hearing loss^6,7^. With ~1–1.6% of individuals in the general population carrying a heterozygous *STRC* deletion^8–10^, the estimated incidence of *STRC*-associated hearing loss is 1 in 16,000^9^. Numerous studies suggest that DFNB16 constitutes a significant proportion of the genetically heterogeneous SNHL, and *STRC* is increasingly recognized as one of the most significant contributors to autosomal recessive nonsyndromic SNHL.

*STRC* contains 29 exons encompassing approximately 19 kb and is tandemly duplicated with the coding sequence of the second copy, the pseudo-*STRC* (*pSTRC*) gene^4^. The first half of the functional *STRC* copy is 100% identical to *pSTRC*. Over the entire locus, *STRC* and *pSTRC* are 98.9% homologous across exons and introns^7^. Therefore, pathogenic SNVs identified by targeted sequences across the entire *STRC* coding region should always be differentiated from those derived from the amplification products of *pSTRC*. Conventional direct PCR and short-read massively parallel sequencing (MPS) are unable to provide consistently reliable data on *STRC*. In fact, some variants previously reported as pathogenic likely reflect codetection with pseudogenes rather than causal mutations in the *STRC* coding sequence^4^. In this study, we present rapid, cost-effective, and precise methods for the diagnosis of *STRC*-related hearing loss by ddPCR and long-range PCR (LR-PCR).

## Methods

### Patients

We analyzed archival DNA samples from 84 unrelated Japanese patients (51 females and 33 males; age range, 5–77 years; mean ± standard deviation, 40.02 ± 18.5 years) with mild-to-moderate SNHL who visited the Department of Otolaryngology at Tokyo Medical and Dental University Medical Hospital between April 2000 and September 2010 and who were diagnosed with idiopathic or autosomal recessive nonsyndromic bilateral SNHL^11^. None of the patients were from consanguineous families. A total of 107 Japanese individuals with normal hearing were included as controls. Based on Sanger sequencing results, we previously excluded pathogenic mutations in the coding sequences of *GJB2* and *Mt-1555*^11^. This study was approved by the research ethics committee of Tokyo Medical and Dental University.

### Screening of CNVs with ddPCR

**Fig. 1:**
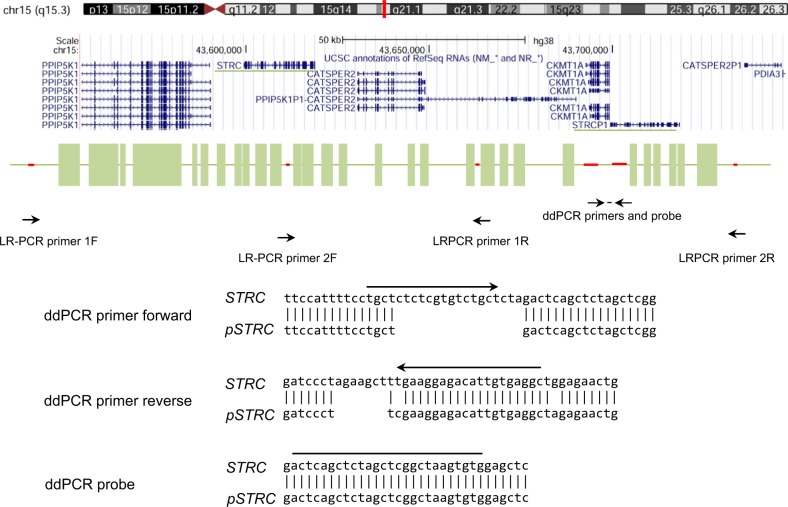
Schematic diagram for the organization of *STRC*, *pSTRC*, and the positions of each primer and probe. The upper part of the figure shows the genomic locations across multiple genes. In the lower part of the figure, green boxes indicate exons of *STRC* with 99% identity to *pSTRC*. Red lines indicate sequences unique to *STRC*; primers were designed to amplify *STRC* but not *pSTRC*

DNA integrity and quality were verified by a NanoDrop spectrophotometer and agarose gel electrophoresis. The ratio of absorbances at 260 nm and 280 nm for each DNA sample was ≥1.8, and all samples were diluted at a concentration of 10 ng/μl by pure water. CNVs were detected by ddPCR with standard protocols using the QX100^TM^ ddPCR system (Bio-Rad, Hercules, CA). Briefly, the master mix for ddPCR included 1 × ddPCR supermix for probes (no dUTP; Bio-Rad), 1.0 μM primer, and 0.25 μM probe (Integrated DNA Technologies, Skokie, IL, USA), together with 20 ng sample DNA. Primers and a probe were designed for intron 23 of *STRC* (Fig. 1, Table 1). This region was chosen as one of the few regions of the *STRC* genomic locus with base pairs that differ from those of *pSTRC* and therefore allows for unique probe sequences. To define the extent of *STRC* CNVs, additional primers and probes recognizing approximately 10-kbp upstream and 39-kbp downstream sequences of *STRC* were also designed (Fig. 1, Table 1). Primers and a probe were also designed for *RPP30* (Fig. 1, Table 1) as the internal control. A black hole quencher was used in combination with the fluorescent dye reporters FAM and HEX. The samples were thoroughly mixed and transferred to a DG8 cartridge for a QX100^TM^/QX200 droplet generator (Bio-Rad). Droplet generation oil for probes (Bio-Rad) was then added to the cartridge, which was placed into the QX200 Droplet Generator^TM^ (Bio-Rad). After droplet generation, the droplets were carefully transferred to a twin-tec semi-skirted 96-well PCR plate (Eppendorf AG, Hamburg, Germany), and the plate was sealed for 5 s at 180 °C using a PX1 PCR plate sealer (Bio-Rad). Subsequent amplification was performed in a C1000 Touch thermal cycler (Bio-Rad) with a ramp rate of 2 °C/sec. First, the enzyme was activated at 95 °C for 10 min, followed by 40 cycles of denaturation at 94 °C for 30 s and 55 °C for 1 min. The enzyme was deactivated at 98 °C (10 min), and the reaction was held at 4 °C. Droplets were read using a QX200 droplet reader ^TM^ (Bio-Rad), and the ddPCR data were analyzed using the Quantasoft software program version 1.7.4 (Bio-Rad). Manual thresholds were applied for both *STRC* and *RPP30*.

**Table 1:** ddPCR primer and probe sequences used in this study

Assay Set	Type	Sequence
*STRC*	Forward Primer	TGCTCTCTCGTGTCTGCT
	Probe	ACTCAGCTCTAGCTCGGCTAAGTGTG
	Reverse Primer	GCCTCACAATGTCTCCTTCA
*STRC* down	Forward Primer	TGAGCAGCCAATGCTACAG
	Probe	TG + CC + CTAA + G + T + TTA + TG
	Reverse Primer	AGGAGAGCCCACCTATTTCT
*STRC* up	Forward Primer	TTCCTCCAACACTACACAGATG
	Probe	TTCCCTCCCTAGCTCCCTTCCTTT
	Reverse Primer	AGGGAGAGAGAGAGAGAGAGA
*RPP30*	Forward Primer	GATTTGGACCTGCGAGCG
	Probe	TTCTGACCTGAAGGCTCTGCG
	Reverse Primer	GCGGCTGTCTCCACAAGT

+ Indicates LVA modification to increase the Tm of the probe and provide greater probe specificity

### LR-PCR-based *STRC* sequencing

To exclude pseudogene sequences, two LR-PCR products were generated for the subsequent nested PCR. Sequences of primers that specifically amplify *STRC* but not *pSTRC* were obtained from the literature^7,12^. LR-PCR was performed with the Qiagen Long-Range PCR Kit (Qiagen, Hilden, Germany) using cycling profiles. Amplification products were loaded and run on a 0.6% agarose gel, and the fragments of appropriate DNA sizes were extracted from the gel using NucleoSpin® gel and a PCR clean-up kit (Macherey-Nagel, Dueren, Germany). Nested PCRs and sequencing continued after LR-PCR products for a portion of *STRC* exon 16, which were used for verification of amplification from only the *STRC* sequence. PCR products were purified using a QIAquick PCR Purification Kit (Qiagen) and were directly sequenced using the Applied Biosystems Prism BigDye Terminator Cycle Sequencing Ready Kit and an ABI Prism 3130xl Genetic Analyzer.

## Results

### *STRC* CNVs and SNVs

**Fig. 2:**
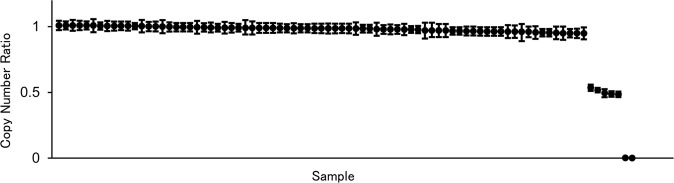
Copy number variation in *STRC* in patients with mild-to-moderate hearing loss. All *STRC*/*RPP30* copy number ratios are arranged in descending order and classified into groups centered at approximately 1.00, 0.50, or 0.00. Each group reflects preservation (no deletion), loss of one copy, or loss of two copies of *STRC*. The error bars for each sample represent the 95% confidence intervals using Poisson statistics

First, we performed ddPCR to identify *STRC* CNVs in 84 patients with SNHL and 107 healthy controls. Among the patients with SNHL, the *STRC*/*RPP30* copy number ratio ranged from 0.949 to 1.009 (0.989 ± 0.017) in 77 patients and from 0.484 to 0.538 (0.509 ± 0.024) in five patients; the ratio was 0.000 in two patients. The ratios of 0.509 and 0.000 likely indicated heterozygous and homozygous deletions, respectively (Fig. 2). Accordingly, seven of the 84 patients carried at least one *STRC* copy loss, which was detected in only one of the 107 healthy controls as a heterozygous deletion (Table 2). The samples from those individuals with *STRC* deletions showed the same deletion pattern by both the 10-kbp upstream and 39-kbp downstream probes. The prevalence of copy number deletion among the patients with mild-to-moderate hearing loss was significantly higher than that among the healthy controls (7.7% vs. 0.9%, *p* < 0.05, Student’s *t* test).

**Table 2:** The prevalence of *STRC* deletion in patients and healthy controls in a Japanese cohort

	Cases	Deletions
Healthy controls	107	1
Patients with SNHL	84	7

**Fig. 3:**
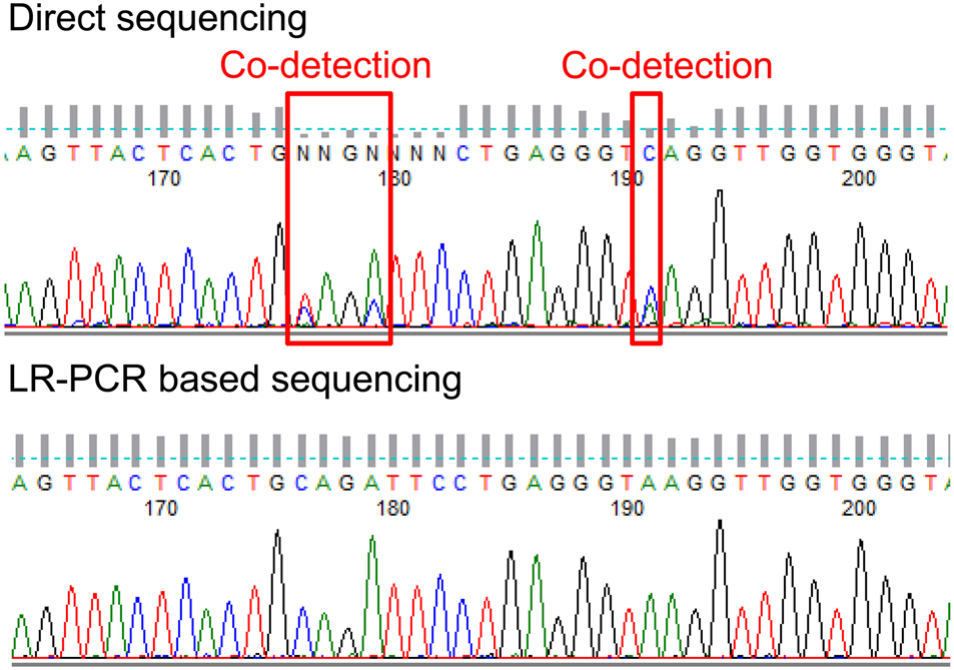
Long-range PCR amplification of *STRC* eliminates codetection with pseudogenes. Direct Sanger sequencing using standard PCR and long-range (LR)-PCR products. Double peaks in the standard PCR-based sequencing trace indicate codetection with a pseudogene, which is eliminated in the sequencing trace of the LR-PCR product

Next, we performed LR-PCR-based *STRC* sequencing in five patients carrying the heterozygous *STRC* deletion. LR-PCR-based sequencing successfully detected SNVs without *pSTRC* contamination, which was observed in conventional direct sequencing (Fig. 3). LR-PCR-based sequencing revealed six types of SNVs in the exons and the untranslated regions of *STRC* in the opposite allele (Table 3). Among these, the c.4057 C > T mutation detected in case 3 was previously reported as a pathogenic SNV resulting in a stop codon. The pathogenicity of c.−28 C > G remained uncertain, while the remaining four SNVs were synonymous substitutions. Overall, three cases (cases 1, 2, and 3) among the study cohort were considered to exhibit *STRC*-related hearing loss.

**Table 3:** Clinical features of the patients with *STRC* deletion

Case	Age^a^/ Sex	Onset (yr)	Averaged hearing level^b^ (right/left)	Progression of hearing loss	Vertigo	Allele 1	Allele 2
1	5/F	5	36.0/40.0	undetermined	No	Deletion	Deletion
2	26/F	10	42.0/40.0	Yes	No	Deletion	Deletion
3	22/F	6	38.0/38.0	Yes	Yes	Deletion	c.446 T > C, c.4057 C > T
4	32/F	25	50.0/45.0	Yes	Yes	Deletion	c.-28 C > G, c.179 T > C, c.446 T > C c.4842 C > T, c.4878 C > G
5	34/M	17	49.0/48.0	Yes	No	Deletion	c.446 T > C
6	54/F	53	62.0/62.0	undetermined	No	Deletion	No variation
7	43/F	33	39.0/34.0	Yes	No	Deletion	No variation

^a^Age at initial visit

^b^Averaged hearing level means arithmetic value of thresholds in 0.5, 1, 2, and 4 kHz

### Clinical and audiologic findings

**Fig. 4:**
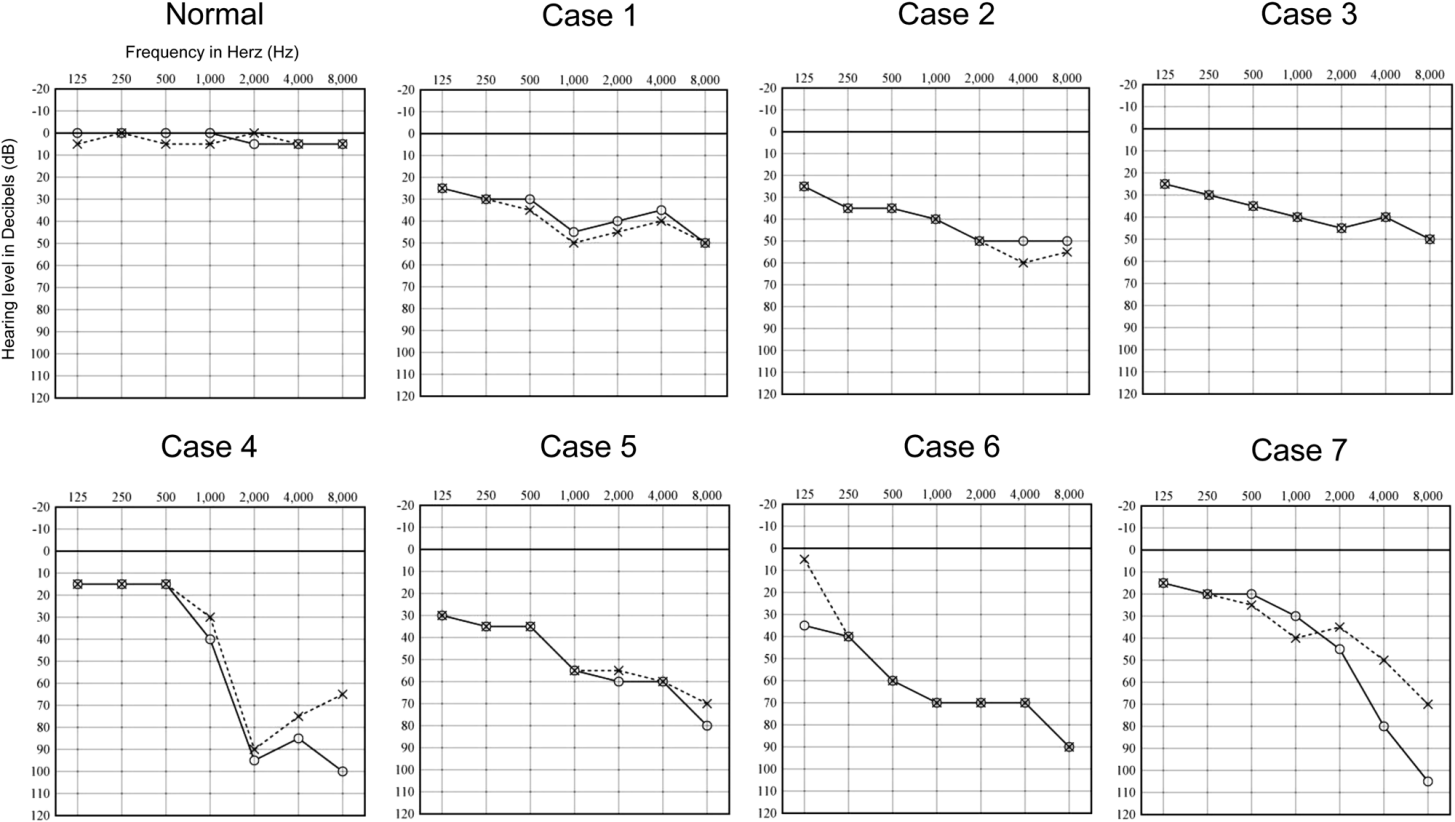
Bilateral pure tone audiograms of patients with hearing loss associated with *STRC* mutations. The vertical axis represents the hearing level, and the horizontal axis represents the frequency. Circles and crosses in audiograms represent the right and left ears, respectively. A typical normal audiogram shows hearing thresholds of both ears above the 20 dB line for all frequencies. All audiograms of patients show mild-to-moderate, horizontal, or high frequency hearing loss

Clinical findings and audiograms of the patients with at least one *STRC* copy loss are summarized in Table 3 and Fig. 4, respectively. The ascertained onset of hearing loss varied from the first to the sixth decade of life, and five patients complained of hearing loss progression. The severity of hearing loss ranged from mild to moderate, and the audiograms showed symmetrical flat or high frequency SNHL. Episodes of vertigo were reported by two of the seven patients. None of the patients exhibited overt family histories and were recognized as solitary cases.

## Discussion

We evaluated the CNVs and SNVs in Japanese patients with mild-to-moderate SNHL using ddPCR in combination with LR-PCR. The prevalence of copy number deletion was 7.7% among the patients. In addition to the homozygous deletion of *STRC* in two patients, LR-PCR-based *STRC* sequencing detected six distinct SNVs in the exons and the untranslated regions of *STRC* in patients with heterozygous *STRC* deletions. One of the SNVs was previously reported as pathogenic^13^. Therefore, at least three patients (3.6%) were considered to exhibit hearing loss associated with *STRC* dysfunction. All patients were recognized as sporadic cases.

Recent studies indicate that approximately two-thirds of the entire human genome is composed of repeats^14^, and 4.8%–9.5% of the human genome can be classified as CNVs^15^. Among these, large repeats, such as whole gene repeats, are generally detected and identified by fluorescent in situ hybridization, array comparative genomic hybridization, single nucleotide polymorphism or oligo arrays, and paired-end mapping. Recently, MPS has also been used to detect CNVs with high genomic resolution. Indeed, MPS is used in the clinic and can efficiently detect CNVs in most Japanese patients with hearing loss^2,10^. These techniques are suitable for searching the whole genome at once but require a considerable amount of work, time and cost. Conversely, ddPCR is insufficient for global identification; however, it provides rapid and easy completion at less than $10 per sample, and the total assay time is 2.5 h, illustrating its efficacy in determining the copy number of specific DNA areas^16^. Additionally, the results are highly reproducible, with minimal variability among DNA samples that does not require strict sample preparation and that is accurate and acceptable for homogenous and heterogeneous statuses. Considering the relatively high prevalence of *STRC* deletion in the Japanese population and challenges in diagnosing inheritable causes of hearing loss, screening with ddPCR is a potential approach before evaluation of patients by MPS.

As a causal gene for mild-to-moderate idiopathic SNHL, *STRC* is garnering increasing attention. Our results reconfirm that CNVs in *STRC* are a common phenomenon in mild-to-moderate SNHL, found in more than 7% of the patients with a carrier rate of less than 1% in healthy Japanese controls, which is consistent with previous reports^2,10^. However, no mutations in the exons of the translated regions of the opposite allele were detected in four of the five patients with heterozygous gene deletion. It is possible that these patients might harbor mutations in genes other than *STRC*, such as *GJB2* and *Mt-1555*. Alternatively, the significantly high prevalence of CNVs in the patients compared to the healthy controls suggests undetectable mutations that might be present in the intronic, promoter, or enhancer regions of *STRC*. Further studies are warranted to verify this hypothesis.

Numerous pathogenic SNVs across the entire *STRC* coding sequence have been reported thus far. However, direct PCR or MPS cannot distinguish whether such variants originate from *STRC* or *pSTRC*. Indeed, the c.4057 C > T, Q1353X variant is coincident with the reference sequence for exon 20 from the *pSTRC*^17^. Therefore, novel nonsense variants that are detected in patients with hearing loss should be verified to ensure that they are a pathogenic variant in the *STRC* coding sequence and not a pseudogene contamination. We recommend LR-PCR-based sequencing as an excellent technique that might be necessary for comprehensive screening for *STRC*-related hearing loss.

In conclusion, *STRC* copy number deletion was detected in approximately 1% of the Japanese healthy controls, whereas *STRC* was the causal gene in more than 3% of patients with mild-to-moderate hearing loss in Japan. The detection of CNVs using ddPCR is a rapid, precise, and cost-effective method for relatively prevalent deafness genes, such as *STRC*. The combination of ddPCR and LR-PCR-based sequencing is a reliable approach to detect patients with *STRC-*associated hearing loss.
